# Mucosal Inflammatory Response to *Salmonella typhimurium* Infection

**DOI:** 10.3389/fimmu.2014.00311

**Published:** 2014-07-04

**Authors:** Samir Patel, Beth A. McCormick

**Affiliations:** ^1^Department of Microbiology and Physiological Systems, University of Massachusetts Medical School, Worcester, MA, USA

**Keywords:** *Salmonella typhimurium*, mucins, microbiota, epithelial barrier, immune recognition, neutrophil recruitment, mucosal inflammation, PMN transmigration

## Abstract

The human intestinal epithelium consists of a single layer of epithelial cells that forms a barrier against food antigens and the resident microbiota within the lumen. This delicately balanced organ functions in a highly sophisticated manner to uphold the fidelity of the intestinal epithelium and to eliminate pathogenic microorganisms. On the luminal side, this barrier is fortified by a thick mucus layer, and on the serosal side exists the lamina propria containing a resident population of immune cells. Pathogens that are able to breach this barrier disrupt the healthy epithelial lining by interfering with the regulatory mechanisms that govern the normal balance of intestinal architecture and function. This disruption results in a coordinated innate immune response deployed to eliminate the intruder that includes the release of antimicrobial peptides, activation of pattern-recognition receptors, and recruitment of a variety of immune cells. In the case of *Salmonella enterica* serovar typhimurium (*S. typhimurium*) infection, induction of an inflammatory response has been linked to its virulence mechanism, the type III secretion system (T3SS). The T3SS secretes protein effectors that exploit the host’s cell biology to facilitate bacterial entry and intracellular survival, and to modulate the host immune response. As the role of the intestinal epithelium in initiating an immune response has been increasingly realized, this review will highlight recent research that details progress made in understanding mechanisms underlying the mucosal inflammatory response to *Salmonella* infection, and how such inflammatory responses impact pathogenic fitness of this organism.

## Introduction

*Salmonella enterica* serovar typhimurium (*S. typhimurium*) is a Gram-negative, facultative, intracellular anaerobe that causes severe inflammation of the intestinal mucosal epithelium resulting in gastroenteritis. *S. typhimurium* causes disease through its primary virulence mechanism, the type III secretion system (T3SS). There are two T3SSs that are encoded by two regions of the bacterial chromosome called *Salmonella* pathogenicity island 1 and *Salmonella* pathogenicity island 2 (SPI-1 and SPI-2). These pathogenicity islands also encode effector proteins that are secreted from the T3SS and translocated into epithelial cells at the mucosal surface of the intestine. Upon contact with the mucosal epithelium, SPI-1 encoded effector proteins are translocated into epithelial cells and promote bacterial entry and inflammation. SPI-2 encoded effector proteins generally function to maintain the intracellular survival of *S. typhimurium* after the organism has been macropinocytosed by epithelial cells. More recent studies, however, suggest that SPI-1 and SPI-2 effector proteins may not be as functionally compartmentalized as originally thought (1–3).

**Figure 1:**
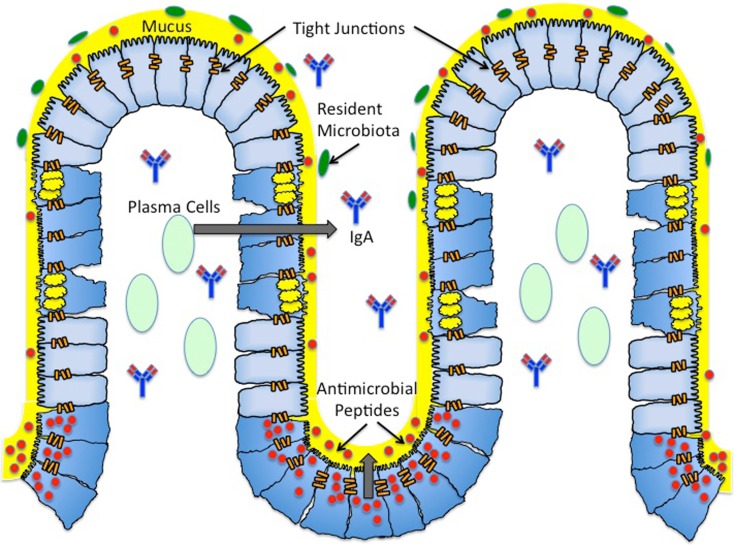
**Architecture of the mucosal surface**. The mucosal surface of the intestine contains a single layer of epithelial cells. The monolayer of epithelial cells is fortified by a layer of mucus (yellow) produced by *Goblet cells* (*blue cells with yellow granules*). This thick mucus layer contains membrane bound and secreted mucins. The antimicrobial peptides (red) secreted by *Paneth cells* (*blue cells with red granules*) reside in the thick mucus layer, providing another form of protection against both pathogenic and commensal bacteria. Antimicrobial peptides include defensins, cathelicidins, and histatins. *Plasma B cells* (*light green*) reside in the subepithelial region and produce *secretory IgA* (*blue and red antibody*). Secreted IgA is found in the subepithelial region and the lumen. *Resident microbiota* (*green*) reside in the outer mucus layer, providing yet another barrier to pathogenic infection. The majority of resident microbiota belong to two phyla – *Firmicutes* and *Cytophaga*–*Flavobacterium* – *Bacteroidetes*. The seal between epithelial cells is maintained by *tight junctions* (*orange bars*). Tight junctions are dynamic structures composed of zonula occludens and junctional adhesion molecules.

The architecture of the mucosal epithelium contains several barriers that attempt to prevent or impede infection by pathogenic bacteria. Mechanisms of protection are employed by all of these barriers in order to maintain the integrity of the epithelial cell monolayer and limit inflammation-associated damage (Figure 1). *S. typhimurium* can modulate the signaling pathways that govern these mechanisms, including targeting specific proteins or inducing pathways through functional mimicry, in order to provide itself with an ecological advantage with its T3SS virulence mechanism. Although *S. typhimurium* can, in certain instances, bypass the innate immune response, the adaptive inflammatory immune response is in most instances capable of clearing the pathogen, albeit with increased damage to the mucosal epithelium.

## Architecture of the Mucosal Epithelium: Barriers Against Infection

### Mucus/mucins

The luminal side of the intestinal epithelium is covered with a thick layer of mucus primarily composed of mucins, the main secretory product of goblet cells (Figure 1). Mucins are high molecular weight glycoproteins that aggregate to form a “gel-like” barrier to defend against endogenous or exogenous luminal insults. To date, at least 17 highly conserved mucins have been identified, each with varying specificities for different epithelial tissues [for review, see Ref. (4)]. Furthermore, these mucins have been categorized into two major groups: cell surface mucins and secreted mucins (Figure 1). Of these two categories, it is the secreted mucins that form the major structural component of the mucosal layer, and out of the known secreted mucin proteins, MUC2, MUC5AC, MUC5B, MUC6, and MUC19 are classified as gel-forming for human mucosal surfaces [for review, see Ref. (5, 6)]. The predominant mucin comprising the mucus layer of the intestinal epithelium is MUC2, although MUC5AC has been shown to be expressed in the mucus layer of the fetal intestine (7).

The mucosal layer consists of an inner layer of mucus that is firmly adherent to the intestinal epithelial cells (mainly comprised of cell surface mucins) and a looser outer layer of mucus (mainly comprised of secreted mucins) [for review, see Ref. (8)]. For quite some time, the mucus layer of epithelial surfaces was thought to solely serve the purpose of providing a physical barrier, preventing access of pathogenic bacteria or resident microbiota to the epithelial cells. However, it has been increasingly realized that the mucins in the outer sublayer of the mucosal barrier also provide an energy source for both resident microbiota and pathogenic microorganisms capable of adhering to the mucus layer. This layer provides both commensal and pathogenic microorganisms with a niche in which to grow and colonize the intestine [Ref. (9); for review, see Ref. (10)]. The inner layer of the mucosal surface is considered “sterile,” largely due to the presence of antimicrobial peptides secreted by Paneth cells (Figure 1, discussed later), thereby limiting bacterial colonization to the outer mucus layer [Ref. (8); for review, see Ref. (11, 12)].

Certain cell surface mucins in the inner mucus layer also directly play a role in protecting against bacterial colonization on the epithelial surface by acting as pathogen-binding decoys. For example, epithelial cells can release Muc1 (called mucin shedding) in response to *Helicobacter pylori* infection, and Muc1 will bind the bacteria, preventing its adhesion to the intestinal epithelium (8). Furthermore, it has been shown that approximately fivefold more *H. pylori* colonize the intestinal epithelium of *Muc1^−^*^/−^ mice than wild-type mice (13). Although the thick mucus layer provides protection in the form of a physical barrier, it is significant to note that the necessity to maintain healthy intestinal microflora does provide pathogenic bacteria with the same energy source and corresponding growth advantage as well. This advantage has allowed certain pathogenic bacteria to develop mechanisms to circumvent the protection provided by the mucus sublayers and infect the underlying epithelial cells. As an example, certain pathogenic *Escherichia coli* (*E. coli*) secrete mucinolytic proteins, thus allowing them to persist and colonize within the mucus layer (14, 15). Unlike *E. coli, S. typhimurium* does not enzymatically degrade mucus in order to colonize the mucosal epithelium. Rather, mucins have actually been shown to be the binding sites for *S. typhimurium*, and in particular a 250-kDa neutral mucin has been implicated as a receptor for *S. typhimurium* (9).

### Resident microbiota

The mammalian intestinal microflora contains ~10^14^ resident bacteria, comprising ~1,000 species, and they reside in the outer sublayer of the mucosal barrier on the luminal side of the intestinal epithelium (Figure 1). The vast majority (~90%) of the commensal bacteria in humans and mice belong to two phyla: *Firmicutes* and *Cytophaga*–*Flavobacterium*–*Bacteroidetes*. Though much of the resident microbiota are of the same two phyla, there are differences in intestinal floral composition of individuals that arise at the species level (16). Diversity of the intestinal microflora is susceptible to change due to environmental factors such as nutrition, and there is variation (increases/decreases in quantity of certain species of bacteria or increases/decreases in diversity of a particular genus of bacteria) in microbiota populations within different age groups (17, 18).

The resident microbiota promote resistance to infection by pathogenic microorganisms in several ways. First, they serve as a microbial barrier by competing with pathogens for resources at the outer mucosal sublayer, thereby limiting pathogenic bacterial colonization (8). Additionally, end products of metabolic pathways of individual species of bacteria have been shown to prevent pathogenic infection. For example, *Bifidobacteria* carbohydrate metabolism produces high concentrations of acetate, which has been shown to prevent release of Shiga toxin during infection with enterohemorrhagic (EHEC), thereby decreasing the risk of toxin gaining access to the blood stream from where it can otherwise cause lethal damage to target organs such as the kidneys (19). Along the same lines, it is becoming more appreciated that the composition of the intestinal microbiota may be just as important in defending against infection as the quantity of the commensal bacteria. For instance, selective reduction of *Lactobacilli* and *Enterococci*/group D streptococci groups of bacteria through the use of low concentrations of antibiotics has been shown to make mice more susceptible to colonization of the epithelial surface with *S. typhimurium* without drastically affecting the overall numbers of commensal bacteria (16). Further investigation is needed to determine exactly which resident microbiota are necessary to prevent other pathogens from colonizing the intestinal epithelium, especially since certain enteric pathogens have developed mechanisms to subvert this microbial form of protection.

Nevertheless, an emerging concept is that inflammation of the mucosal epithelium plays a role in the bacterial fitness of *S. typhimurium*. One of the more basic advantages of *S. typhimurium*-induced inflammation is that the clinical manifestation of diarrhea facilitates the spread of bacteria. Additionally, it has been shown that unlike avirulent strains, wild-type *S. typhimurium* is capable of out-competing commensal microbiota in re-colonization experiments after treatment with antibiotics. Furthermore, *S. typhimurium* exploits inflammation to promote its own colonization. In this instance, *S. typhimurium* has been shown to out-compete the resident microbiota in a mouse colitis model (20). One explanation for this phenomenon is that inflammation provides *S. typhimurium* with a respiratory electron acceptor that members of the resident microbiota are unable to utilize. In particular, reactive oxygen species generated by neutrophils (PMNs) during inflammation can react with endogenous thiosulfate to form tetrathionate, a respiratory electron acceptor (21). The ability to respire tetrathionate has been mapped to the *ttrRSBCA* locus, which is located in SPI-2 (22). Under anaerobic conditions in which thiosulphate was oxidized to tetrathionate, *S. typhimurium* displays a growth advantage in comparison to resident microbiota under the same conditions (21).

Both resident microbiota and *S. typhimurium* compete for resources available for fermentation at the mucosal layer; however, resident microbiota are incapable of using the fermentation end products (21). By reducing the tetrathionate made available by the inflammatory response to infection, *S. typhimurium* is capable of respiring the fermentation end products in anaerobic conditions, thereby providing it with an advantage over the resident microbiota (21). Remarkably, the growth benefit is conferred to *S. typhimurium* only in the presence of inflammation, and it has been suggested that a reason *S. typhimurium* has evolutionarily maintained its inflammation-inducing virulence mechanisms could be to provide it with an ecological advantage at the mucosal surface of the intestine (21).

### Paneth cells

Paneth cells are specialized epithelial cells located at the base of crypts of Lieberkuhn that generate and secrete antimicrobial peptides of ~20–40 amino acids in length (Figure 1). There are four families of antimicrobial peptides: defensins, cathelicidins, histatins, and lactoferrin (Figure 1, Table 1). Defensins are positively charged and directly interact with the negatively charged membrane of pathogenic microorganisms resulting in membrane destabilization and pore formation. Cathelicidins are also positively charged, and they function in binding and neutralizing lipopolysaccharides (LPS), ultimately resulting in pore formation. Unlike defensins and cathelicidins, histatins do not interact with the membranes of pathogenic bacteria. Instead, histatins are ingested by the bacteria, inhibit mitochondrial respiration, and kill the microorganism by generating reactive oxygen species [for review, see Ref. (23)]. Lactoferrin is a cationic protein that sequesters iron, an essential nutrient for pathogenic microorganisms. Additionally, lactoferrin can bind LPS and destabilize bacterial membranes similar to defensins and cathelicidins (23, 24). The antimicrobial activities of these peptides are non-specific, as their activity provides a first line of defense against both Gram-positive and Gram-negative bacteria, fungi, and enveloped viruses.

**Table 1:** **Summary of antimicrobial peptides**.

Antimicrobial peptides	Function	Reference
Defensins (i.e., HD-5, HD-6)	Destabilization of bacterial membranes	(23, 25, 26)
Cathelicidins (i.e., CRAMP, LL-37)	Neutralization of LPS	(23, 27, 28)
Histatins	Generation of reactive oxygen species	(23)
Lactoferrins	Sequestration of iron and destabilization of bacterial membranes	(23, 24)

All antimicrobial peptides are produced in an inactive, pre-propeptide form and must be processed (i.e., enzymatically) either intracellularly or extracellularly to become active (10). For example, the alpha-defensin HD-5 is stored in Paneth cells in an inactive, pre-propeptide form and is processed by trypsin into its active form (29). Antimicrobial peptide production has been shown to be upregulated in response to bacteria (30). However, pathogenic microorganisms have developed methods to counteract the effectiveness of the antimicrobial peptides. Examples of these methods include covalently modifying the bacterial cell membrane to reduce its net negative charge, using bacterial proteases to catalytically inactivate the antimicrobial peptides, and using ATP-driven pumps to physically remove the antimicrobial peptides from the bacterial cytoplasm [for review, see Ref. (31)]. Certain pathogens have developed resistance to the antimicrobial activities of the peptides secreted by Paneth cells in order to promote their intracellular survival.

Antimicrobial peptides that provide protection against *S. typhimurium* infection have been identified using transgenic mouse models. Alpha-defensin HD-5 transgenic mice were shown to consistently have a significant reduction in the *S. typhimurium* burden in the distal intestine and spleen in comparison to wild-type mice that do not express this antimicrobial peptide, indicating the antimicrobial activity of HD-5 conferred the transgenic mice with an enhanced ability to kill *S. typhimurium* in the intestinal lumen (26). Another alpha-defensin shown to provide increased defense against *S. typhimurium* infection is HD-6, which binds bacterial membrane proteins, thereby inhibiting contact of *S. typhimurium* with epithelial cells (25). Since HD-6 does not kill *S. typhimurium*, HD-6 transgenic mice do not display the decrease in bacterial burden seen with HD-5 transgenic mice; however, HD-6 transgenic mice display a profound increase in survival rate in comparison to wild-type mice that do not express this antimicrobial peptide, indicating the antimicrobial activity of HD-6 must act in concert with another defense mechanisms at the mucosal barrier to eliminate *S. typhimurium* (25).

In addition to defensins, mouse models have also identified the significance of cathelicidins and lactoferrin. The sole murine cathelicidin called cathelin-related antimicrobial peptide (CRAMP) has been shown to impair intracellular replication of *S. typhimurium in vivo* and *in vitro* (27). Additionally, *S. typhimurium* displayed enhanced survival in macrophages derived from CRAMP-deficient mice (27). CRAMP is similar in structure and antimicrobial properties to the only human cathelicidin called LL-37, which has been shown to display a broad spectrum of activity against bacteria including *S. typhimurium* (28). A recent study identified the *in vivo* effect of lactoferrin on *S. typhimurium*. In this study, mice treated with bovine lactoferrin displayed a reduction in severity, mortality, and inflammation during infection, indicating the antimicrobial properties of lactoferrin are significant for defense against *S. typhimurium* (24).

### The epithelial barrier

In addition to mucosal defenses described above, interactions between cells of the epithelial cells in the monolayer also provide a barrier against bacterial entry. Tight junctions are dynamic structures composed of zonula occludens (ZO) and junctional adhesion molecules that effectively adhere the cells of the epithelial monolayer to each other (Figure 1) (8). The integrity of this seal is maintained by the interaction of tight junction components with the actin cytoskeleton. However, the permeability of this seal is regulated by physiological conditions, and it therefore can be manipulated. For example, treating epithelial monolayers with inflammatory cytokines, such as IL-1β, increases the permeability of the tight junctions (32). The increase in tight junction permeability can facilitate the translocation of bacteria from the lumen to the subepithelial region, making them a target for pathogenic manipulation. Pathogenic microorganisms can accomplish the manipulation of tight junctions by usurping signaling pathways, such as the Rho-GTPase pathway, which regulates actin cytoskeleton rearrangement (8).

*Salmonella typhimurium* infection has been shown to regulate certain tight junction proteins, which ultimately promotes translocation of the bacteria through the epithelial cell monolayer (33). Upon infection with *S. typhimurium*, occludin becomes dephosphorylated and subsequently removed from epithelial tight junctions (33). Additionally, ZO-2 is recruited from the cytosol to membrane, indicating *S. typhimurium* alters the intracellular distribution of this tight junction protein (33). Surprisingly, ZO-1, which is normally regulated by pathogens in a similar manner to ZO-2, appears to be degraded during *S. typhimurium* infection (33). Manipulation of tight junction proteins serves to disrupt the epithelial barrier by increasing its permeability, thereby allowing *S. typhimurium* to more effectively invade the basolateral side of the epithelial cell monolayer.

In order to mount a successful infection, *S. typhimurium* must disrupt some aspects of the protective mechanisms employed by the mucosal epithelium. As mentioned previously, the two T3SS and the secreted bacterial effector proteins promote entry, inflammation, and intracellular survival. In addition, in order to subvert the action of antimicrobial peptides, *S. typhimurium* uses the two-component system PhoQ/PhoP, which regulates the expression of SPI-2 encoded genes as well. Specifically, PhoP/PhoQ regulators promote remodeling of the bacterial envelope, resulting in increased resistance to antimicrobial peptides that recognize LPS. Furthermore, the PhoP/PhoQ regulators repress transcription of genes for the T3SS, in attempt to avoid detection, and induce protective mechanisms against hydrogen peroxide (10). In addition to rearranging the actin cytoskeleton and targeting specific tight junction proteins, *S. typhimurium* also manipulates tight junctions via the action of SipA, SopE, SopE2, and SopB (8). These effector proteins induce Rho-GTPase activation, and inhibition of this effector-induced Rho-GTPase activation prevents tight junction disruption (8).

**Figure 2:**
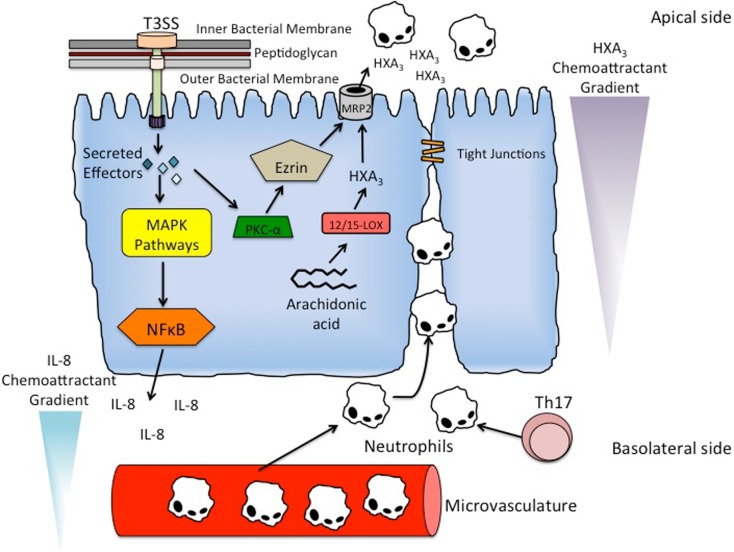
**Mechanism of PMN recruitment and PMN transmigration**. *S. typhimurium* utilizes its T3SS to secrete effector proteins into epithelial cells to activate inflammatory signaling pathways. In particular, the activation of Rho-GTPases by SopE, SopE2, and SopB result in the induction of mitogen-activated protein kinase (MAPK) pathways. The stimulated pathways include ERK, JNK, and p38, resulting in the terminal activation of major inflammatory regulator NF-κB. Activation of NF-κB results in the basolateral secretion of IL-8 producing a chemoattractant gradient that recruits neutrophils to the subepithelial region from the underlying microvasculature. Th17 cells are also present in the subepithelial region, and function to recruit and activate neutrophils in the subepithelium. PMN transmigration is facilitated by another chemattractant HXA_3_. HXA_3_ is a bioactive eicosanoid that is synthesized from arachidonic acid via the 12/15-lipoxygenase pathway in epithelial cells. It is secreted into the lumen via the action of an ATP-binding cassette transporter called MRP2. *S. typhimurium* effector protein SipA stimulates the recruitment of PKC-α to the apical membrane, which in addition to the ERM protein ezrin, modulate the localization of MRP2 to the apical membrane, thereby allowing secretion of HXA_3_ into the lumen and production of the chemoattractant gradient that induces PMN transmigration.

The manipulation of tight junctions has also recently been shown to facilitate the transmigration of PMNs across the epithelial cell monolayer (33). The primary mechanism of PMN migration in *S. typhimurium* infection involves the recruitment of neutrophils into the subepithelium and the formation of a chemoattractant gradient that directs the neutrophils into the lumen (Figure 2, discussed in detail later). However, recent research implicates the disruption of tight junctions in facilitating PMN migration even in the absence of the chemoattractant gradient (33). Thus, *S. typhimurium* not only modulates the release of neutrophil chemoattractants that induce PMN migration, but also directly influences the tight junctions that maintain the fidelity of the epithelial cell monolayer in order to promote bacterial translocation and PMN transepithelial migration.

### Lamina propria

The lamina propria is the connective tissue underlying the epithelial cell monolayer. It contains multiple immune effector cells including B cells, T-cells, dendritic cells, natural killer (NK) cells, macrophages, eosinophils, and mast cells. If enteric pathogens are capable of surmounting the barriers described above and penetrate the intestinal epithelium, a coordinated immune response utilizing these immune effector cells is activated. Sampling of luminal antigens occurs in specialized cells called M cells, which transport the antigens to a subepithelial region where the antigen comes in contact with dendritic cells. Dendritic cells bound to antigen then migrate to the mesenteric lymph node to present the luminal antigens to naïve T-cells and B cells. These naïve lymphocytes then differentiate into several effector cells including CD8 cytotoxic T-cells, CD4 helper T-cells, regulatory T-cells, and antibody secreting B cells. Although this marks the beginning of a coordinated immune response to pathogenic bacteria, the resting lamina propria does have protective functions that provide an added layer of defense prior to the full activation of the mucosal immune system.

The most abundant B cell found in the lamina propria is the IgA-secreting B cell (Figure 1). Secreted IgA is also the primary secreted immunoglobulin found in the thick mucus layer. One of the main roles of secreted IgA is a process called immune exclusion, which includes prevention of pathogens from adhering to the mucosal surface on the luminal side of the intestinal epithelium and removal of antigens from the basolateral side of the intestinal epithelium. On the luminal side, secreted IgA primarily interferes with microbial adhesins, whereas on the basolateral side secreted IgA functions in an export mechanism by binding antigens and shuttling them back across the epithelial monolayer into the lumen (34). Secreted IgA does not activate an inflammatory immune response when it neutralizes pathogenic microorganisms, thereby upholding the integrity while preventing inflammation-induced damage of the mucosal epithelium (35).

Secreted IgA also has a direct effect on the virulence mechanisms of certain pathogens. In *S. typhimurium* infection, it has been shown that a monoclonal, polymeric IgA antibody Sal4 binds the O-antigen (O-Ag) component of LPS on the bacterial membrane, resulting in its destabilization [Ref. (36); for review, see Ref. (37)]. Recent evidence indicates that the bacterial membrane destabilization results in impaired T3SS translocon formation, decrease in effector protein delivery, and decrease in flagellum-based motility (36). *S. typhimurium* responds to the binding of Sal4 to O-Ag by triggering exopolysaccharide (EPS) production and biofilm formation, though this response renders the bacteria non-invasive and avirulent (38). The mechanism of EPS production and biofilm formation has been attributed to the activation of a cyclic dimeric guanosine monophosphate-dependent pathway via an inner membrane diguanylate cyclase YeaJ (38). Furthermore, it has been suggested that the triggering of this pathway by *S. typhimurium* could be a mechanism to restore membrane stability, as EPS production could serve to shed IgA antibody or increase resistance to other luminal insults (38).

The resting lamina propria contains a heterogeneous population of CD8 cytotoxic T-cells, CD4 helper T-cells, and regulator T-cells even in the absence of pathogenic infection. The number of effector T-cells in the resting lamina propria would in any other tissue indicate an inflammatory response; however, the amount of T-cells present in the mucosal tissue of the gut is more indicative of constant immune surveillance and recognition than chronic inflammation. The cytokines produced by these T-cells maintain the mutualistic response to resident microbiota, stimulate production of IgA, induce secretion of antimicrobial peptides, and promote epithelial repair. Additionally, in the absence of pro-inflammatory cytokines (which are usually produced by innate immune cells in the presence of pathogen), the dendritic cells in the resting lamina propria contribute to maintaining tolerance to non-pathogenic antigens by promoting the production of CD4 regulatory T-cells. Regulatory T-cells produce immunosuppressive cytokines that inhibit T-cell proliferation and dendritic cell differentiation, which prevents unnecessary immune response to innocuous antigens (35).

In addition to a heterogeneous population of T-cells, the macrophages in the resting lamina propria play a key role in host defense against *S. typhimurium* infection, as well. The macrophages of the resting lamina propria can be divided into two different classes: M1 (“classically activated”), and M2 (“alternatively activated”) polarized macrophages. M1 polarized macrophages are pro-inflammatory and display high phagocytic and antimicrobial activity, whereas M2 polarized macrophages are anti-inflammatory and display low phagocytic and antimicrobial activity. Hence, with this type of opposing macrophage regulation it is considered that M1 macrophages function in the clearance of infection versus M2 macrophages that assist in wound healing and suppression of T-cell function. Manipulation of macrophage polarization by *S. typhimurium* has become increasingly realized as a defense mechanism against bacterial clearance [for review, see Ref. (39, 40)]. A recent study demonstrated that the SPI-1 T3SS enables *S. typhimurium* to guide macrophage toward the M2 polarization (40). This type of control permits *S. typhimurium* to escape the more hostile environment of M1 polarized macrophages, resulting in a macrophage-specific decrease in pro-inflammatory signaling (40).

The mechanism of immunity to invasive *Salmonella* is still disputed, specifically the relevance of cell-mediated versus humoral immunity. The debate is complicated by attempts to compare different experimental models, which vary in route of *Salmonella* administration and/or susceptibility of mouse strains to *Salmonella*. In terms of cellular immunity, mice deficient for TCR α/β, MHC class II, or interferon-γ (IFN-γ) receptor fail to clear a primary *Salmonella* infection that can be resolved in normal mice (41, 42). Recently, it has also been shown that Thy1^+^ NK cells are essential for the early production of IFN-γ during control of *Salmonella* infection (43). CD8^+^ T-cells seem to also play a role in *Salmonella* clearance (44). It has also been documented that *Salmonella* infection promotes the expansion of intestinal intraepithelial lymphocytes (iIELs) and the activation of particularly, CD8^+^ TCRγδ^+^ iIELs, which in turn trigger cytolytic activity against *Salmonella-*infected epithelial cells (44).

The role of antibody-producing immune cells or B cells against *Salmonella* is still controversial (45–47). Some reports have shown the importance of antibody production and T-cell activation for protection from virulent *Salmonella* (45, 46). However, another study reported that the protective immunity provided by an attenuated *S. typhimurium* strain required B cells independently of antibody production, proposing that they confer protective immunity by presenting antigen to T-cells and acting as a source of inflammatory cytokines (47). It has also been demonstrated that transfer of immune serum into B cell-deficient mice can partially but not completely provide protective immunity (48).

## The Type III Secretion System: Co-Opting Host Pathways to Promote Entry and Immune Evasion

Upon contact of *S. typhimurium* with the epithelial cell monolayer, the SPI-1 effector proteins SopE, SopE2, and SopB initiate the process of bacterial entry by activating host cell Rho-GTPases resulting in actin rearrangements (49, 50). SipA is another SPI-1 effector protein that antagonizes actin depolymerizing agents and tethers actin monomers together to form membrane ruffles, which promotes bacterial internalization (51). *S. typhimurium* is engulfed by epithelial cells through a macropinocytosis event termed bacterial-mediated endocytosis, and is ultimately contained within in a membrane-bound vesicle called a macropinosome [more commonly termed the *Salmonella* containing vacuole (SCV)]. Although prior studies thought that SopB was the sole mediator of macropinosome formation, a cooperative interaction regulated by the phosphatase activity of SopB has implicated SopD as another mediator of this process (49).

*Salmonella typhimurium* also targets antigen-sampling microfold (M) cells to translocate across the gut epithelium. M cells constitute a small subset of highly specialized follicle-associated epithelium (FAE) enterocytes overlying lymphoid follicles in the gut, and are characterized by an irregular brush border, a reduced glycocalyx and lysosomal apparatus, and are programed to efficiently transcytose a wide variety of macromolecules and microorganisms from the gut lumen to the underlying immune inductive Peyer’s patches (PPs) (52). Recent evidence shows the *S. typhimurium* type III effector protein SopB also induces an epithelial–mesenchymal transition of the FAE into M cells. This cellular transdifferentiation is a result of SopB-dependent activation of Wnt/β-catenin signaling leading to induction of both receptor activator of NF-κB ligand (RANKL) and its receptor RANK. The autocrine activation of RelB-expressing FAE enterocytes by RANKL/RANK induces the EMT-regulating transcription factor Slug that marks epithelial transdifferentiation into M cells. Thus, *S. typhimurium* may also transform primed epithelial cells into M cells to promote host colonization and invasion (52, 53).

Following bacterial entry of mucosal epithelia, *S. typhimurium* employs a second set of SPI-1 effector proteins to ensure repair of the actin cytoskeleton. SptP is one such effector that is directly responsible for reversing the affects of SopE and SopE2. SptP promotes restoration of the epithelial cell membrane by functioning as a GTPase-activating protein for the Rho-GTPase proteins Rac-1 and Cdc42 (54). Similarly, as several of the early SPI-1 effectors induce inflammation of the mucosal epithelium, there are effector proteins that have an anti-inflammatory function, providing *S. typhimurium* with a form of regulatory control over the inflammatory state of the mucosal tissue (inflammation induced by *S. typhimurium* will be discussed later).

After successful entry into epithelial cells and restoration of the epithelial cell membrane, *S. typhimurium* relies primarily on the T3SS encoded by SPI-2 to survive and replicate intracellularly by translocating SPI-2 effector proteins across the membrane of the SCV into the epithelial cell cytoplasm. SPI-2 effector proteins that appear to be necessary for survival and virulence of *S. typhimurium* inside the SCV are SifA, SseJ, SseF, SseG, SopD2, and PipB2 (50, 55, 56). SifA has been shown to promote tubulation of the SCV through correlation with another effector protein SseJ (57). SCV tubulation in conjunction with the effects of SseF and SseG localize the SCV to the perinuclear region in close proximity of the Golgi apparatus (58). The localization of the SCV is important for intracellular survival because vesicular trafficking through the Golgi network allows for the acquisition of nutrients, thereby allowing the establishment of a replication niche for *Salmonella* (58, 59).

An additional means by which SPI-2 promotes intracellular survival of *S. typhimurium* is by encoding factors that mediate the evasion of immune responses. SPI-2 promotes protection from reactive oxygen intermediates produced by macrophages, specifically nitric oxide (NO) and NADPH oxidase [for review, see Ref. (60–62)]. *S. typhimurium* has been shown to evade NO-mediated killing in macrophages by inhibiting IFN-γ-induced NO production in a SPI-2-dependent manner (61). SPI-2 is also involved in avoiding NADPH oxidase-dependent killing by interfering with the trafficking of NADPH oxidase (62). Although the specific SPI-2 effector proteins involved in the evasion of both NO-dependent and NADPH oxidase-dependent killing of *S. typhimurium* have yet to be identified, the established role of SPI-2 in evasion of both immune responses suggests a possible role for one or more encoded effector proteins in promoting resistance to reactive oxygen intermediates in macrophages.

## *Salmonella*-Induced Inflammation

### Immune recognition

Pathogen-associated molecular patterns (PAMPs) are recognized by pattern-recognition receptors (PRRs), namely toll-like receptors (TLRs), located on inflammatory cells and epithelial cells. TLRs can recognize a wide range of PAMPs, though some TLRs do show some specificity for particular PAMPs. For example, TLR4 is mostly involved in recognition of LPS and TLR5 is mostly involved in the recognition of bacterial flagellin. TLRs in epithelial cells are localized to the basolateral or apical membrane, as well as in intracellular vesicles. Thus, TLRs can recognize pathogens on either side of the epithelial cell monolayer and endocytosed extracellular pathogens. Additionally, inflammatory cells, such as macrophages, expressing TLRs can also recognize PAMPs. The importance of some TLRs, specifically TLR4 and TLR5, in *S. typhimurium* infection have been established, as mutating them has been shown to increase susceptibility to infection and inflammation (35). Intracellular recognition of bacteria or their products in the cytoplasm is also mediated by nucleotide-binding oligomerization domain proteins NOD1 and NOD2. NOD1 recognizes peptides containing diaminopimelic acid, which is a component of Gram-negative bacterial cell walls, whereas NOD2 recognizes a muramyl dipeptide present in the peptidoclycan layers of both Gram-positive and Gram-negative bacteria. Similar to TLRs, mutations in NOD1 and NOD2 proteins increase susceptibility to disease and infection caused by intracellular bacteria [for review, see Ref. (63, 64)].

### Recruitment of immune cells

The host immune system also activates inflammatory pathways in response to infection with *S. typhimurium*. The binding of TLRs and NOD1/NOD2 proteins to their respective ligands activates the NF-kB pathway leading to production of pro-inflammatory cytokines and chemokines. Basolateral secretion of the cytokine IL-8 recruits neutrophils and is necessary for PMN migration into the subepithelium. Additional chemokines, such as CCL20, play a role in attracting immature dendritic cells, which upon exposure to antigen, can mature and present antigenic peptides to naïve B and T-cells in the mesenteric lymph nodes (35). *S. typhimurium* can also react with TLRs on macrophages in the subepithelial region after being transcytosed through M cells, thereby activating and inducing them to also produce cytokines and chemokines. Cytokines produced by these activated macrophages include IL-1, IL-6, and IL-23, all of which drive the differentiation of T_H_17 cells whose primary function in the subepithelium is recruiting and activating neutrophils (Figure 2) (35, 65). Other cytokines produced by these activated macrophages include IL-18 and IL-12, both of which drive the IFN-gamma-dependent production of antigen-specific T_H_1 cells (35).

### Mechanism of neutrophil recruitment

A hallmark of *S. typhimurium*-induced inflammation is the recruitment of PMNs from the underlying microvasculature to the subepithelial region of the epithelial cell monolayer (Figure 2). The neutrophils then migrate across the monolayer into the lumen, resulting in the inflammatory pathology of Salmonellosis. New information is shedding light on the molecular mechanisms and signaling pathways involved in neutrophil recruitment across the intestinal epithelium. As discussed above, it is becoming increasingly appreciated how inflammation induced by *S. typhimurium* increases its pathogenic bacterial fitness.

In addition to promoting bacterial entry, many effector proteins encoded by SPI-1 also activate inflammatory signaling pathways. The activation of Rho-GTPases by SopE, SopE2, and SopB result in the induction of mitogen-activated protein kinase (MAPK) pathways (Figure 2). In particular, the ERK, JNK, and p38 pathways are stimulated, resulting in the terminal activation of inflammatory regulators AP-1 and NF-κB (Figure 2) [Ref. (66); for review, see Ref. (50)]. Furthermore, the activation of NF-κB and AP-1 stimulates the secretion of the cytokine IL-8 on the serosal side of the epithelial cell monolayer, a requirement for the recruitment of neutrophils to the subepithelial region (Figure 2) (67). Although IL-8 is necessary for PMN migration into the lumen, it has been shown that IL-8 alone is not sufficient enough to drive the migration across the epithelial cell monolayer [Ref. (67, 68); for review, see Ref. (69)].

The migration of neutrophils from the basolateral side to the luminal side of the epithelial cell monolayer is driven by another PMN chemoattractant, hepoxilin A_3_ (HXA_3_) (Figure 2) (68, 70). HXA_3_ is a bioactive eicosanoid that is synthesized from arachidonic acid via the 12/15-lipoxygenase pathway in epithelial cells (Figure 2) (70). After synthesis, HXA_3_ is secreted from the apical surface of epithelial cells by an ATP-binding cassette transporter called multidrug resistant protein 2 (MRP2) (Figure 2) (71). Secretion of HXA_3_ into the lumen forms a chemoattractant gradient that causes neutrophils to migrate from the region underlying the epithelial cell monolayer into the lumen (Figure 2) (70).

Activation of the effector protein SipA has been shown to be necessary for induction of HXA_3_ synthesis and the resulting PMN migration (Figure 2) (72). Remarkably, the mechanism for activating SipA was recently shown to require processing by the host enzyme caspase-3 at a particular cleavage site, resulting in two distinct effector domains (73). Furthermore, the two domains were shown to be functionally different. The ability to promote PMN migration is confined to the SipA N-terminal domain, whereas the C-terminal domain has been shown to be involved in actin rearrangement (72, 73). The current understanding of the mechanism of SipA-dependent synthesis of HXA_3_ is that SipA induces the recruitment of ADP-ribosylation factor 6 (ARF6) to the apical membrane of the epithelial cells. ARF6 activates phospholipase D, which generates phosphatidic acid. Phosphatidic acid is then converted to diacylglycerol (DAG), which recruits protein kinase C-α (PKC-α) to the apical membrane (Figure 2). PKC-α, in addition to an ERM protein ezrin, modulate the localization of MRP2 to the apical membrane of epithelial cells, thereby allowing the secretion of HXA_3_ into the lumen and production of the chemattractant gradient that induces neutrophil transmigration (Figure 2) (72, 74, 75).

## Conclusion

The architecture of the mucosal immune system, including mucins, antimicrobial peptides, resident microbiota, paracellular junctions, and effector cells of the lamina propia, functions to prevent pathogenic bacteria from disrupting the epithelial cell monolayer and causing disease. If enteric pathogens are able to penetrate these barriers, then it results in a host inflammatory response and eventually activation of an adaptive immune response, designed to eradicate the intruding pathogen. However, *S. typhimurium* has evolved systems, namely the SPI-1 and SPI-2 T3SS, to manipulate the defensive mechanisms of the mucosal immune system in order to develop a replication niche in the mucosal epithelium. Additionally, the ability of *S. typhimurium* to exploit inflammation allows it to penetrate the epithelial barriers, a condition in which activation of the adaptive immune response would be required for pathogenic clearance. Investigating how *S. typhimurium* exploits host cell signaling pathways will allow for increased understanding in its pathogenesis, and consequently provide further insight into how inflammation can seemingly result in both increased bacterial fitness and increased pathogenic clearance.

## Conflict of Interest Statement

The authors declare that the research was conducted in the absence of any commercial or financial relationships that could be construed as a potential conflict of interest.
